# Arrested and Deficient Lactation

**Published:** 1885-04

**Authors:** Henry F. Campbell

**Affiliations:** Augusta, Georgia


### ARRESTED AND DEFICIENT LACTATION.

BY HENRY F. CAMPBELL, M. D., AUGUSTA, GEORGIA.

A failure in the adequate supply of milk for the proper and entire nourishment of the infant is, in modern times, a not infrequent occurrence. Such deficiency is always disappointing to the mother and often calamitous to the child, by impairment of its nutrition, as artificial substitutes cannot be made in all cases to subserve the ends of the ordained and natural nutriment.

For such deficiency, existing in a greater or less degree, tonics, malt liquors, nourishing diet and sometimes an oppressive amount of fluids, with perhaps warm and stimulating applications to the mammæ, are well known to be the staple resources of both the medical attendant and the experienced nurse.

My own experience, as that of most practitioners, supplies a large number of deficient milk-supply, treated with varying results by these ordinary methods. Among my notes, however, there are two or three cases, one quite recent and others some years ago, in which the arrest of lactation was sudden and complete, after what had appeared to be a very satisfactory and full establishment of the flow. The essential features of these cases I will briefly state, as the results obtained may prove of practical interest.

Case I. Mrs. V. J. M., aged twenty years, in second child-bcd, September 22, 1875. Her primapara had been with forceps—living child—lactation imperfect, the child sustained by the bottle, was feeble and died in dentition. This second parturition natural, and there was a slight milk flow on the third day, continued scantily for one week, child being fed with the bottle. In another week the milk ceased entirely. Galvano-faradic mild current applied before bed time—one pole at nuclea and circle completed by hand on breasts for twenty minutes. The milk-flow became abundant during the night—"clothing drenched" by the spontaneous flows. The milk continued for a few days, but in uncertain quantities, and then ceased, to the bitter disappointment of the mother. The battery was again applied repeatedly, but causing no adequate supply, the patient became discouraged, and it was abandoned, as the child was doing well on the bottle.

Case II. Mrs. R. M. W., aged twenty-three years, was in her third child-bed in 1878. Labor had been easy and natural. Lactation was fully and rather profusely established on the third day, and continued abundant for over a week. On one of my morning visits I was informed that she thought her milk had ceased to flow, that it had sensibly diminished for the past two days, and that now none could be obtained, and the child was being fed from the bottle. The breasts were, both of them flabby, and both areolæ pale. The lochia were natural and she felt no particular discomfort in the pelvic region. She was without fever, but had lost her appetite entirely, and had a depressed and melancholy look, and complained of slight frontal headache, the bowels and kidneys acting naturally; urine not tested. Mrs. W. had drunk freely of table tea and other fluids, hoping to recover her milk-flow, without any result whatever.

I directed warm fomentations to the mammæ and a weak sinapism to dorsal and cervical spine, with milk punch and continuance of the fluids. Finding no change on my call next day, and being at that time in frequent application of the battery to certain cases, I proposed the application of a mild electric current. Though somewhat startled at the proposition, she willingly submitted.

The ~~galvano-~~faradic current was made in the following manner: One pole of the battery—my notes do not record which—was held in the hand of the patient and the other in my own right hand, while with the left I completed the connection by alternately passing the hand over the two mammæ. The strength of the current was low, but could be gradually increased without complaint from the patient. It was continued for twenty minutes. She stated that "she had a more natural feeling in her breast now than she had had for several days," and she evidently *expected* much from the unusual procedure.

On calling the day after the application, she requested that I would apply the battery again, and stated that she had felt "the springing of milk" in her breasts, and nursed the child and thought he had sucked a little milk. The application was made as before. A full flow of milk returned during the night. On the following day the breasts were fully distended, and to the end of lactation, there was no variation from the full and abundant supply which had marked her first nursing periods and that of her children born subsequently.

Case III. Mrs. B. E. F., aged 21—first birth January 24th, 1885. Natural; no incidents during parturition; lactation established on third day in normal manner; nipple not well developed; and child nursing imperfectly, the breast became painfully distended. The breast-pump, and then a young puppy, was used to empty the breasts. The full flow continued four days. In one week from the birth of the child the milk began to fail. At the end of the next week it had entirely ceased. The mammary glands were hanging shrunken and soft, at which the lady seemed more annoyed than at the loss of the milk. "Her breasts had never looked that way before in all her life!"

I applied as in the above case the electric current from one of Jerome Kidder's batteries. The application lasted twenty minutes to a mild degree. The patient within an hour remarked that she was not certain, but thought she felt the milk springing in one of her breasts. During the night, twelve hours after, the distention was very decided, and the breast-pump being applied, one ounce or more was drawn and the child applied to the breast. On the day following, the child not nursing sufficiently, the pump had to be twice applied to relieve the painful distention.

The application of the current in this case was made daily by the husband, for one week, during which the secretion continued in full quantity. The use of the battery was then abandoned, when, to the disappointment of all, the milk rapidly failed again, and none was secreted. On its re-application "a decided effect was produced, but not so remarkable as at first." To-day (March 6th) I am informed that the battery, not acting well, has been discontinued for over a week, and that the flow of milk has almost ceased.

In this second case, though not so fortunate in its final results as in case first, the influence of the galvanic current in awakening the milk secretion seemed more marked, as repeated opportunities were afforded for observing the effect of both its application and withholding. Unlike the first case, this lady throughout the entire period continued in good health and spirits, and in every respect her general condition was as comfortable and favorable as could be desired—except in the failure of lactation. I have advised that a mild daily current be again resumed, with dry-cupping to the cervical and dorsal spine, and nourishing fluid diet, in the hope that the secretion may again possibly be aroused.

### THE NEURO-DYNAMIC INFLUENCES AFFECTING THE MAMMARY GLAND AND LACTATION.

It is almost unavoidable, in considering the effect of such an agent as electricity or galvanism on any function, whether that function be sensory, motor, secretory or trophic, that we have suggested to our minds the nervous influences and instrumentalities that are involved, or supposed to be involved, in the *rationale* of all such procedures.

To those who claim—and generally it may perhaps be justly claimed—that for each of the vital acts occurring in any portion of the system there is required a nerve-force of a specific kind and having its source and origin in nerve-centres unvarying in their kind and location; as, for instance, vision recognized in the optic lobes, olfaction with its special centre, pulmonary respiration with the invariable termination of its nervous apparatus centrally in the "respiratory tract" of the spinal cord, while the medulla oblongata has been proved experimentally to influence the glycogenic function of the liver, and to modify the secretion and constituents of the urine (Claude Bernard); and further, more recently, contention is made and largely prevails, that the convolutions can be located (Farrier, Bartholow, Hitzig and others)

on which special muscular acts depend for their dynamic and coördinating influence: "To such," as I had begun to say, the mammary gland and its functional phenomena are a stumbling-block and a confusion. A gland varying little in its histological structure, with a secretory product analogous, at least, if not almost identical throughout the entire class to which it gives its designation, the supreme intent and purpose to which it is appointed, always the same; governed and controlled by direct nervous influences, apparently, as other secretory organs are supposed to be governed, and perhaps more than any one of them, disturbed by *reflex* influences* of the most marked, peculiar and invariable character, and yet *seeming* to derive, so far as we can judge by locality of the gland itself, its nervous supply and its neuro-dynamic excitation and control, from no one particular portion of either brain, spinal cord or ganglionic centre. Not to look through the long line of the mammalians, let us point out a few variations in the location of the gland: In the human female, and others of the higher class, they are double, and located on the upper and front aspect of the thorax; in some quadrupeds, as the horse, the ass, cow, goat, sheep, and many others not so familiar, near the groin and at the pelvic end of the trunk; while in others of this latter class, as the dog, the cat, the rabbit, the sow, and other multiparious animals, the mammary apparatus is multiple and ranged in a double row at intervals from the thoracic to the pelvic end of the trunk. Like cutaneous transpiration and other elementary functions of the skin, then it appears to be a matter of indifference from what particular portion of the central nervous system or region of the spinal cord, the mammary gland and the lacteal secretion derive the neuro-dynamic influence, if, as some may suggest, any such influence whatever controls and

---

*"The intimate relations subsisting between the mammary gland and the uterus, whether non-gravid, gravid or puerperal, are too well recognized and familiar for any extended comment. The breasts develop under the advance of puberty, they enlarge and become tender during menstruation, they color their areolæ after conception; while, during pregnancy and after labor, the relation is even still more striking and mutual. The nursing infant excites painful, but often beneficial, uterine contractions, and the uterine processes seem intimately connected with the establishment of lactation. All the above phenomena, heretofore ignorantly attributed to "sympathy," are now recognized as the result of normal reflexes in the spinal cord."

See Prophylactic and Therapeutic Value of Quinine in Gynectic and Obstetric Practice. By Henry F. Campbell, Amer. Gynecological Transactions, vol. V. Cincinnati, 1881.

directs the process. This innervation and nerve-force seems to be of the simplest kind, and can be no other and not differing from that which controls and governs the functional activities of every kind which are exercised by the general integument of the body.

CONCLUSIONS.

*First.*—That from a consideration of the varying locality of the mammary gland upon the trunk of the several genera of mammalia, the nervous supply being furnished indifferently by any portion of the central spinal system; the object and the efficiency of the secretion being the same in all of them as in man; and especially from the known fact that anomolies in women have transferred the gland to abnormal localities, as the groin, etc., it may be decided that the neuro-dynamic excitation in the mammæ of the human female is of the simplest nature, and no other than that under which the functions of the integument, as sensation and secretion are accomplished.

*Second.*—After the foregoing conclusion in regard to the simplicity of the neuro-dynamic influences concerned in the function of lactation, and in the light of the experience of the cases herein reported, we may reasonably expect the stimulus of a well selected and judiciously applied electric or galvanic current to prove, in many cases of arrested and deficient lactation, a hopeful and often an efficient therapeutic measure.

March 6, 1885.

