# Correction

**Published:** 1885-04

**Authors:** 


Correction.—In the article by Dr. H. F. Campbell, in this issue of the Journal, page 76, third paragraph, first line, for *galvano*-faradic current, read faradic current.

